# Comprehensive Metabolomic and Transcriptomic Analysis Revealed the Molecular Basis of the Effects of Different Refrigeration Durations on the Metabolism of *Agaricus bisporus* Cultivation Spawn

**DOI:** 10.3390/jof11060415

**Published:** 2025-05-27

**Authors:** Zhixin Cai, Zhiheng Zeng, Wenzhi Chen, Zhongjie Guo, Huiqing Zheng, Yuanping Lu, Hui Zeng, Meiyuan Chen

**Affiliations:** Institute of Edible Mushrooms, Fujian Academy of Agricultural Sciences, Fuzhou 350012, China; caizhixin75@163.com (Z.C.); pawnmaker@163.com (Z.Z.); 13123167516@163.com (W.C.); 3515002713@163.com (Z.G.); 18065028416@163.com (H.Z.); yuanplu1106@163.com (Y.L.); zenghui@faas.cn (H.Z.)

**Keywords:** *A. bisporus* cultivation spawn, low-temperature storage, metabolome, transcriptome

## Abstract

*Agaricus bisporus* is popular worldwide because of its high nutritional value and low cost. Low-temperature storage is a common storage method used for the production and sales of *A. bisporus* cultivation spawn, but few studies have focused on the physiological and biochemical mechanisms associated with low-temperature storage of *A*. *bisporus* cultivation spawn. In this study, we examined *A. bisporus* spawn samples stored for different refrigeration periods (0, 20, 40, 60, 80, and 100 days), measured changes in the activities of four key extracellular enzymes and performed transcriptomic and metabolomic analyses. The results of the enzymatic assays revealed that the activities of carboxymethyl cellulase (CMCase), amylase, and acid protease initially decreased before increasing, whereas laccase activity showed the opposite trend. This pattern may represent an energy supply mechanism adopted by *A. bisporus* to cope with low temperatures, where extracellular enzymes indirectly influence survival by mediating substrate decomposition. Further correlation analysis on the basis of CMCase activity changes revealed 148 carboxymethyl cellulase-correlated metabolites (CCMs) and 514 carboxymethyl cellulase-correlated genes (CCGs) (*p* ≤ 0.05), and significance was determined at FDR < 0.05 with a fold change > 1.5. Among these, 56.08% of the CCMs and 63.04% of the CCGs presented positive correlations with CMCase activity, whereas 43.92% and 36.96% presented negative correlations, respectively. Integrated multiomics analysis revealed significant variations in metabolic flux and gene expression across different storage durations. Two CCMs (ketoleucine and 3-methyl-2-oxovaleric acid) gradually decreased in expression, whereas two CCGs (AbbBCAT and AbbAACS) increased in expression. This study provides novel insights into the molecular adaptation of *A. bisporus* spawn to refrigeration, highlighting the importance of branched-chain amino acid metabolism in the cold stress response and storage stability.

## 1. Introduction

*Agaricus bisporus*, commonly known as the white button mushroom, which is the most widely produced and marketed commercial mushroom species globally [1]. The consumption of *A. bisporus* provides numerous health benefits, including hepatoprotective, immune system-enhancing, and cardiovascular protection effects [2,3]. In industrial production, low-temperature storage is the primary method to maintain the viability and marketability of *A. bisporus* cultivation spawn to the greatest extent [4,5]. However, while storage can delay the physiological and biochemical activities of the fungus, excessively long periods at low temperatures accelerate the aging of spores, severely affecting their vitality [4,5]. Once these aging spawns are sold for planting, they can reduce yield or even lead to no harvest, which severely affects the stability of *A. bisporus* cultivation and the economic effects on mushroom farmers. Therefore, understanding the impact of low-temperature storage on the physiological and biochemical processes of *A. bisporus* cultivated spawn during industrial production is crucial for addressing these issues.

Low-temperature storage is a traditional method used for most edible mushroom cultivation spawn. Previous studies have explored the physiological and biochemical effects of low-temperature storage on mushrooms through proteomic and extracellular enzyme analyses [6,7]. During low-temperature storage of *A. bisporus*, the activities of three extracellular enzymes involved in lignin synthesis are affected, significantly increasing mushroom hardness and allowing maintenance of textural properties [6]. To date, research on low-temperature storage of *A. bisporus* has been limited to changes in external quality (e.g., color and texture) or physiological and biochemical parameters (e.g., vitamins and sugars) [8], but the specific molecular mechanisms underlying these changes remain unclear, there are even fewer studies on the cultivated spawn of *A. bisporus*. Due to lack of understanding at the molecular level, the optimal storage duration for *A. bisporus* cultivation spawn is not known, severely hindering the optimization of its production and the supply chain.

The activity of extracellular enzymes can be used to measure the culture medium utilization rate during *A. bisporus* cultivation spawn growth and can reflect mycelial activity. For the cultivation of *A. bisporus* spawn, economically viable waste biomass, usually a mixture of wheat, sorghum, and other substances are used as a culture medium [9,10]. *A. bisporus* cultivated spawn decomposes the culture medium through extracellular enzymes to store nutrients such as sugars for mushroom growth [11]. By measuring the activity of extracellular enzymes, one can assess the physiological state and growth potential of mycelia under low-temperature conditions. Studies have shown that long-term storage of edible fungi at low temperatures maintains or increases the activity of enzymes such as protease and carboxymethyl cellulase, with minimal phenotypic changes observed and biological potential retained after storage [12]. Therefore, studying the activity of extracellular enzymes in cultivated *A. bisporus* spawn at low temperatures is highly beneficial for understanding spore aging. However, research on extracellular enzymes in *A. bisporus* cultivated spawn to date is incomplete, and studies on the molecular mechanisms involved in low-temperature storage are extremely rare.

For these reasons, selecting appropriate methods for studying the low-temperature storage of *A. bisporus* cultivated spawn is crucial. Multiomics, a systemic approach to biological research, can be used to comprehensively analyze the molecular mechanisms underlying the physiological and biochemical impacts of low-temperature storage on cultivated *A. bisporus* spawn [13,14]. Through transcriptomic studies, bioactive compounds with potential anticancer and antioxidant properties in medicinal mushrooms have been identified [15]. The cultivation of the edible mushroom *Hypsizygus marmoreus* has been optimized via low-temperature restriction processes developed on the basis of combined transcriptomic and metabolomic analysis [13].

The molecular mechanisms underlying *A. bisporus* spawn aging during refrigeration remain poorly understood, particularly the interplay between extracellular enzymes, metabolic flux, and gene expression. To address this gap, we hypothesized that prolonged cold storage triggers biphasic changes in extracellular enzyme activities, driven by shifts in branched-chain amino acid metabolism, ultimately compromising spawn viability. By integrating transcriptomic and metabolomic analyses across six refrigeration time points (0–100 days), this study aims not only decipher the dynamic relationship between extracellular enzyme activities and spawn aging but also identify key metabolites and genes linked to cold stress adaptation and provide actionable thresholds for industrial storage based on molecular evidence.

## 2. Materials and Methods

### 2.1. Sample Collection

The *A. bisporus* cultivation spawn (W192) used in this study was provided by the Institute of Edible Mushrooms, Fujian Academy of Agricultural Sciences. The original spawn of *A. bisporus* was inoculated into breathable bags containing culture media (98% wheat grains and 2% light calcium carbonate, sterilized at 126 °C for 2.5 h). It was then cultured at a temperature of 24 °C for 15 days until the mycelia completely covered all the wheat grains, resulting in a breathable bag containing the wheat grain cultivation spawn. This breathable bag containing the wheat grain cultivation spawn was stored at 8 °C, as it balances metabolic suppression and industrial feasibility. Samples of the wheat grain cultivation spawn were taken on days 0, 20, 40, 60, 80, and 100. Each group had three replicates, which were rapidly frozen in liquid nitrogen before being stored at −80 °C.

### 2.2. Detection of Extracellular Enzyme Activity

Extracellular enzyme activity was detected by using products from Beijing Solarbio Technology Co., Ltd. (Beijing, China), namely, the Carboxymethyl Cellulase Activity Detection Kit (Cat: BC2545), Amylase Activity Detection Kit (Cat: BC0615), Acidic Protease Activity Detection Kit (Cat: BC2280), and Laccase Activity Detection Kit (Cat: BC1630). Assays followed the manufacturer’s protocols with minor modifications: the reaction volume was reduced by 25% to accommodate 96-well plates, and the incubation times were optimized for linear detection ranges. Signals were recorded using a SpectraMax M5 microplate reader (Molecular Devices, San Jose, CA, USA) under the following conditions: carboxymethyl cellulase, amylase, and acid protease activities were measured via absorbance at 540 nm (for reducing sugar products) or 440 nm (for protease-cleaved chromogenic substrates). Laccase activity was quantified via fluorescence (excitation/emission: 285/370 nm) using 2,2′-azino-bis(3-ethylbenzothiazoline-6-sulfonic acid (ABTS) as the substrate. Signal parameters: readings were taken every 30 s for 10 min at 25 °C, with a 5 s orbital shake before each measurement. Data were analyzed using SoftMax Pro 7.1 (Molecular Devices) with linear regression of initial reaction rates (R^2^ > 0.98).

### 2.3. Metabolomic Analysis

Frozen samples (50 mg) were manually homogenized in liquid nitrogen using a sterile mortar and pestle, followed by immediate suspension in extraction buffer with 800 μL of ice-cold 80% methanol/water containing 0.1% formic acid to prevent thawing. After vortexing and sonication at 4 °C for 15 min, the proteins were precipitated at −20 °C for 1 h. The supernatant was collected by centrifugation (12,000× *g*, 15 min, 4 °C) and filtered through 0.22 μm membranes. Pooled quality control samples were analyzed every 10 injections to monitor system stability. The metabolites were then extracted from each sample following a previously described protocol (23). Ultrahigh-performance liquid chromatography (UHPLC)-mediated separation was carried out via a Dionex Ultimate 3000 RS UHPLC (Thermo Fisher Scientific, Waltham, MA, USA) instrument equipped with an ACQUITY UPLC HSS T3 column (1.8 μm, 2.1 × 100 mm, 186009468, Waters, Milford, NH, USA) by Oebiotech Company (Shanghai, China). Mass spectrometry was performed via a SCIEX TripleTOF 5600+ system (AB SCIEX, Redwood City, CA, USA) with an electrospray ionization (ESI) source operating in positive/negative ion mode. The flow rate was 0.35 mL/min, and the mobile phases were 0.1% formic acid in water (A) (A117-50, Thermo Fisher Scientific, Waltham, MA, USA) and 0.1% formic acid in acetonitrile (B) (A998-4, Thermo Fisher Scientific, Waltham, MA, USA). The column temperature was set to 45 °C, the autosampler temperature was set to 4 °C, and the injection volume was 5 μL (24, 25).

SCIEX Analyst Workstation software (version 1.6.3) was employed for MRM data acquisition and processing. MS raw data (.wiff) files were converted to the .txt format via msConvert. The R program and database were used for peak detection and annotation (26–28). Commercial databases, including the Kyoto Encyclopedia of Genes and Genomes (KEGG; http://www.kegg.jp; accessed on 27 November 2024) and MetaboAnalyst (https://www.metaboanalyst.ca/; accessed on 30 November 2024), were subsequently used to search for ‘metabolitepathways’ (https://www.genome.jp/kegg/pathway.html, accessed on 2 December 2024). Multivariate statistical analysis methods were used to analyze differences in the data. The significance criteria used were as follows: *p* value from Student’s *t* test < 0.05 and a fold change > 1.5 or <0.67.

### 2.4. RNA-Seq and Analysis

Total RNA from samples was extracted via the TRIzol method (15596026, Invitrogen, CA, USA), and RNA purity was assessed via a NanoDrop spectrophotometer (Implen, CA, USA). RNA integrity was evaluated via an Agilent 2100 Bioanalyzer (Agilent Technologies, CA, USA), and agarose gel electrophoresis was performed to assess RNA degradation. The extracted RNA was sent to Beijing Novogene Co., Ltd., for library construction and sequencing. After the cDNA libraries were constructed, they were pooled, followed by sequencing on an Illumina HiSeq 4000 platform via a PE 150 sequencing strategy (library construction via reagent kits from Illumina, San Diego, CA, USA). Raw data (raw reads) in fastq format were first processed through in-house Perl scripts, and cutadapt and Perl scripts were used to obtain clean data (29). All downstream analyses were based on clean, high-quality data. The gene expression level was calculated via the FPKM method. The DESeq2 method was used for difference analysis. Finally, differentially expressed genes (DEGs) were screened by setting thresholds of |Log2FC| ≥ 1.0 and a false discovery rate (FDR) < 0.05.

### 2.5. Data Processing and Visualization

Data for the extracellular enzymes were plotted as bar graphs via GraphPad Prism 8. Spearman correlation analysis was performed to assess the correlations among the carboxymethyl cellulase data and multiomic analysis results, and correlation coefficients and *p* values were calculated. The website https://www.bioinformatics.com.cn (accessed on 26 April 2025) was used to generate pie charts showing significantly correlated genes and metabolites. An enrichment analysis of significant metabolites was performed via the KEGG metabolic pathway database. Data analysis and visualization were conducted via the website OmicShare tool available at www.omicshare.com/tools (accessed on 26 April 2025). For metabolomics, *p* values were adjusted via Benjamini–Hochberg FDR correction. For RNA-seq, DESeq2 with FDR < 0.05 and |log2FC| ≥ 1 was used. The obtained sequences were compared for homology with registered sequences in GenBank using the ElasticBLAST 1.4.0 program on the NCBI website. Representative sequences with high homology were downloaded and aligned using Clustal Omega 1.2.4 software. A phylogenetic tree was then constructed using the maximum likelihood (ML) method in MEGA 11 software, with branch confidence (bootstrap) tested through 1000 replicates.

## 3. Results

### 3.1. Shifts in Extracellular Enzyme Activity and Multiomic Differences

**Figure 1:**
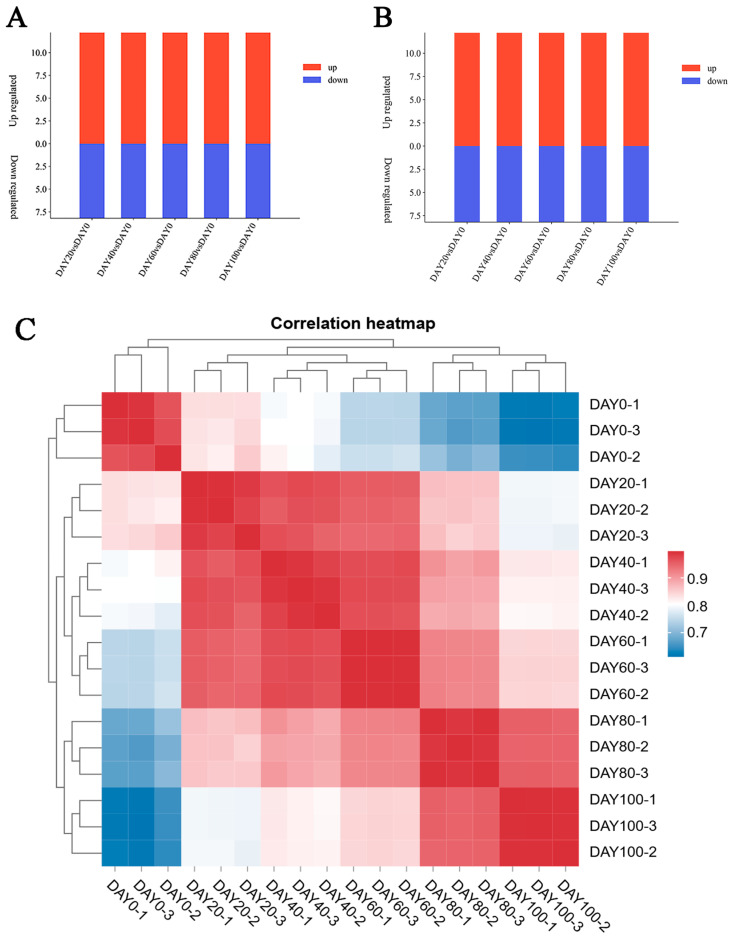

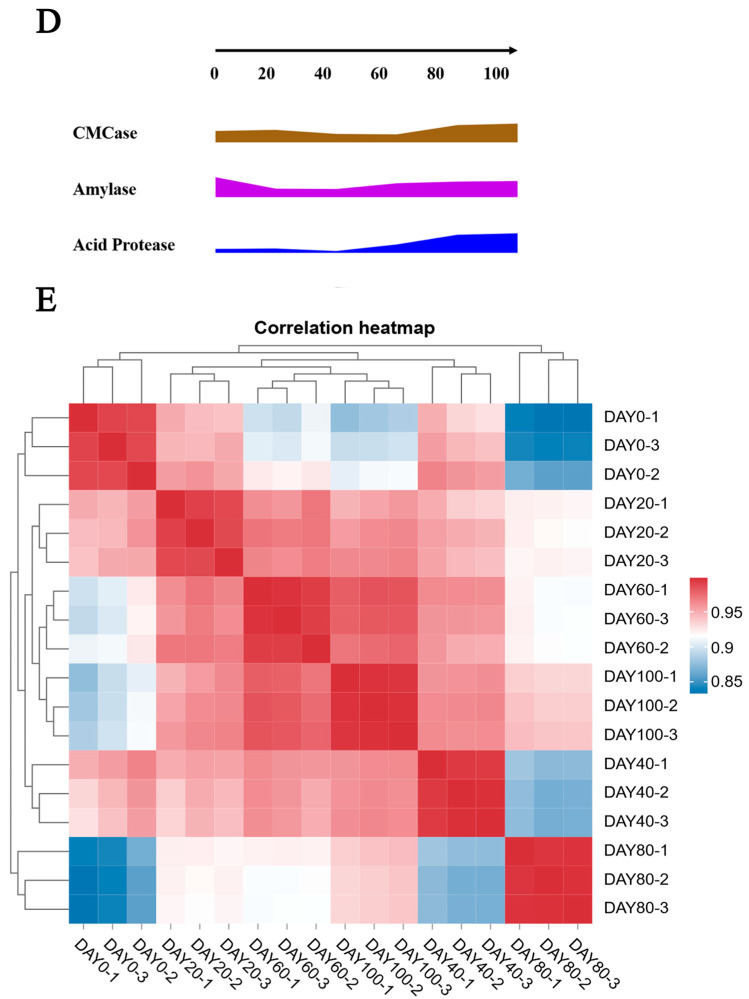
Assessing extracellular enzyme activity and multiomics in cultivated *A. bisporus* spawn under varying refrigeration durations. (**A**) Differentially accumulated metabolite analysis using the day 0 group as a control. Red represents upregulated metabolites, and blue represents downregulated metabolites. (**B**) Differential gene expression analysis was performed using the day 0 group as a control. Red represents upregulated genes, and blue represents downregulated genes. (**C**) Heatmap of gene expression changes at different time points during refrigeration. Gene expression levels were normalized to a zero mean, shown from green to pink, with higher values indicating higher expression levels. (**D**) Activities of 4 extracellular enzymes after different refrigeration durations. (**E**) Heatmap of metabolite changes after different refrigeration durations. The expression levels of the metabolites were normalized to a zero mean, shown from green to pink, with higher values indicating higher expression levels.

A total of 770, 823, 1122, 1338, and 1192 DAMs (Figure 1A) were identified in the comparisons with the day 20, 40, 60, 80, and 100 samples, respectively. And a total of 5501, 5711, 6317, 6156, and 6695 DEGs (Figure 1B) were identified in the comparisons with the day 20, 40, 60, 80, and 100 samples, respectively. RNA-seq analysis was performed to reveal the molecular mechanism underlying the effects of refrigeration on cultivated *A. bisporus* spawn. Figure 1C shows the expression levels of 9822 genes on days 0, 20, 40, 60, 80, and 100. The 0-day sample was used as a control for further DEG analysis. To study the changes in the activities of extracellular enzymes from cultivated *A. bisporus* spawn, we used carboxymethyl cellulase, amylase, acid protease, and laccase enzyme activity detection kits to detect enzyme activity on days 0, 20, 40, 60, 80, and 100. As shown in Figure 1D, on days 0, 20, and 40, the enzyme activities of carboxymethyl cellulase, amylase, and acid protease decreased gradually (*p* < 0.05), and the enzyme activity of laccase increased gradually. On days 60, 80, and 100, the enzyme activities of carboxymethyl cellulase, amylase, and acid protease increased gradually (*p* < 0.05), and the enzyme activity of laccase decreased gradually. This was because the peak activity of carboxymethyl cellulase occurs during the mature stage of the fruiting body, whereas the activity of laccase is relatively high during the mycelial growth stage and decreases with increasing cultivation time [16]. Among the four extracellular enzymes, CMCase was the most representative. To study the changes in metabolites in *A. bisporus* cultivated spawn after different refrigeration durations, a broadly targeted metabolomic technique using a UHPLC system was implemented. A total of 2665 metabolites were detected in the test samples, and these metabolites were identified through UHPLC–MS/MS analysis with reference to standard databases. The expression levels of these genes on days 0, 20, 40, 60, 80, and 100 are shown as heatmaps in Figure 1E. The day 0 sample was used as a control for further differentially abundant metabolite (DAM) analysis.

### 3.2. 148 Cellulase-Correlated Metabolites Identified

**Figure 2:**
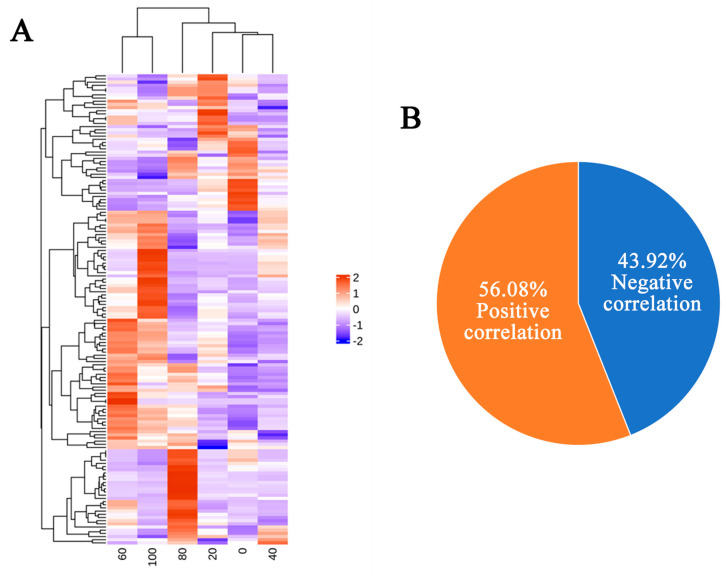

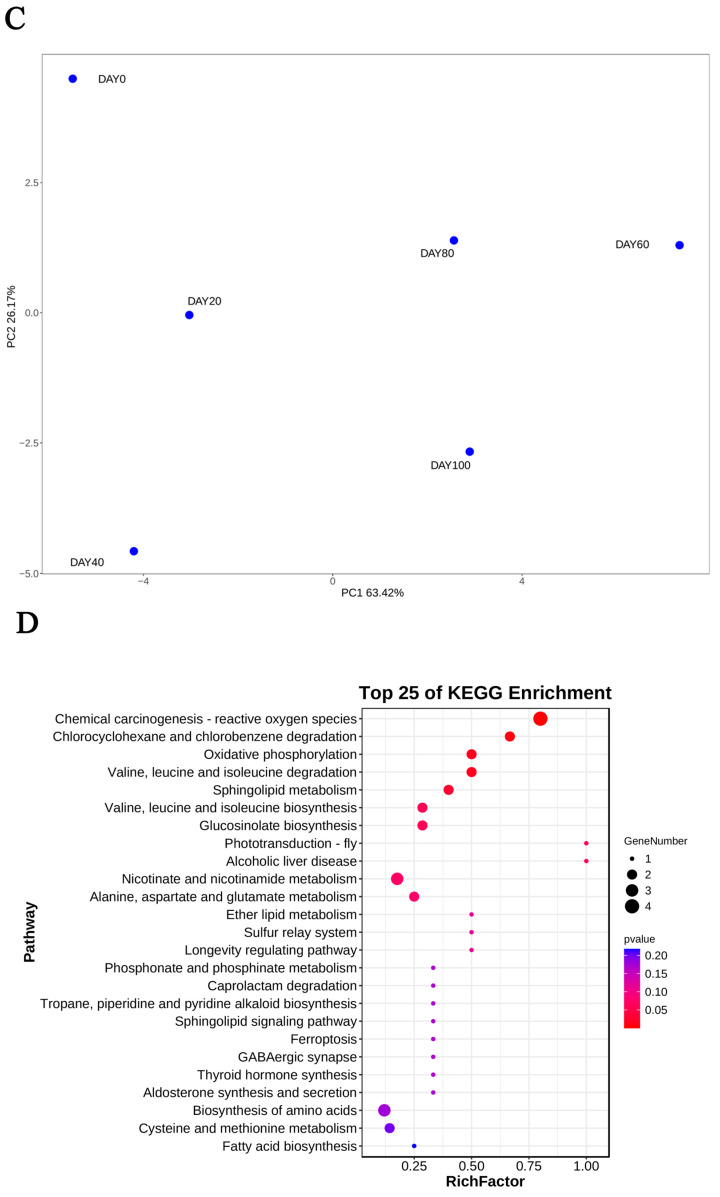
CCMs observed during refrigeration. (**A**) Heatmap of CCMs after different refrigeration durations. The expression levels were normalized to a zero mean, shown from blue to red, with higher values indicating higher expression levels. (**B**) A pie chart displaying the number and proportion of CCMs (*p* < 0.05, based on the Spearman correlation). (**C**) PCA of changes in CCMs after different refrigeration durations. The abscissa represents the first principal component PC1, and the ordinate represents the second principal component PC2. (**D**) KEGG enrichment analysis where the x-axis represents the proportion of CCMs in each pathway out of all CCMs, and the y-axis represents the name of the identified pathway. The size of the circles indicates the number of CCMs in each metabolic pathway, and their color indicates the *p* value from the concentration assay.

To further study the relationship between the changes in extracellular enzyme activity and the metabolome, we performed a Spearman correlation analysis on the changes in CMCase activity and the metabolome data, and a total of 148 carbohydrate cellulase-correlated metabolites were identified (*p* ≤ 0.05). The expression levels of these genes on days 0, 20, 40, 60, 80, and 100 of refrigeration are shown as heatmaps in Figure 2A. Among the 148 carboxymethyl cellulase-correlated metabolites (CCMs), 56.08% were positively correlated (*p* < 0.05) with changes in the activity of CMCase, and 43.92% showed the opposite correlation (*p* < 0.05) (Figure 2B). PCA of the observed metabolite expression levels revealed obvious differences across all six samples (Figure 2C), and all the samples were within the 95% confidence interval. The contribution rate of the first principal component (PC1) was 63.42%, the contribution rate of the second principal component (PC2) was 26.17%, and the sum of the contribution rates of the two principal components was 89.59%. The top 25 metabolic pathways that were found to be significantly enriched by KEGG enrichment analysis of these CCMs are shown in Figure 2D; these pathways included chlorocyclohexane and chlorobenzene degradation, oxidative phosphorylation, and valine, leucine, and isoleucine degradation.

### 3.3. 514 Cellulase-Correlated Genes Detected

**Figure 3:**
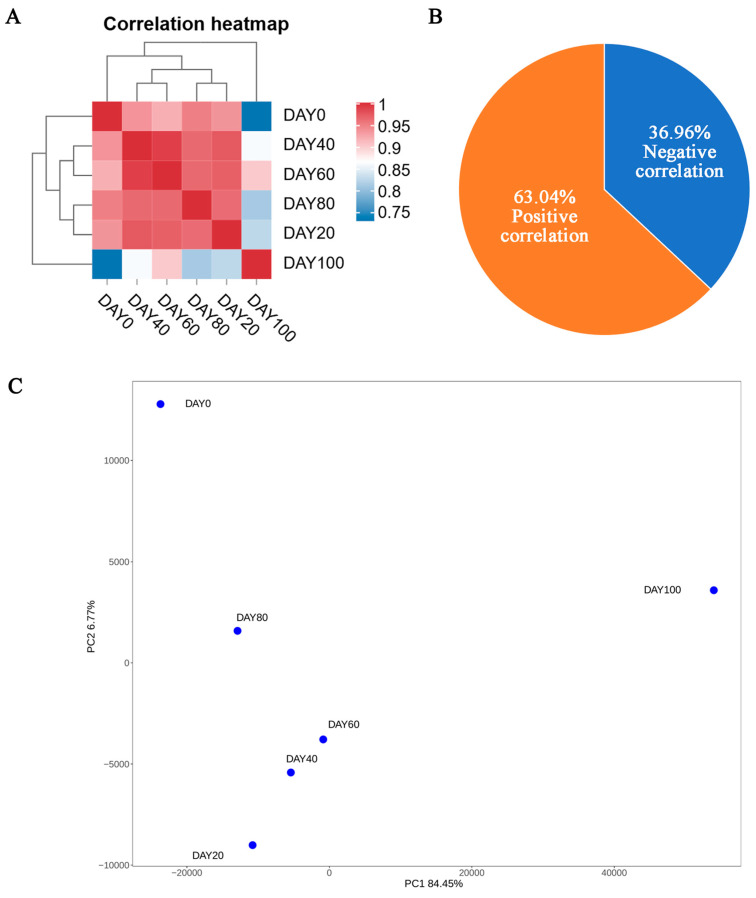

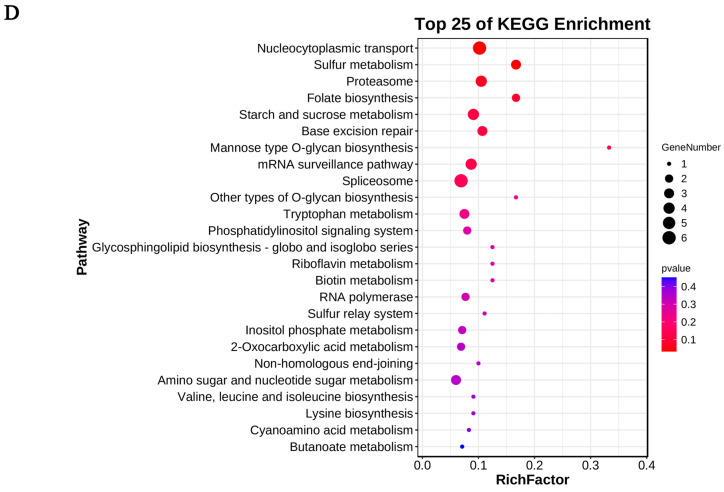
CCGs of *A. bisporus* cultivated spawn during refrigeration. (**A**) Heatmap of CCG changes after different refrigeration durations. Gene expression levels were normalized to a zero mean, shown from blue to red, with higher values indicating higher expression levels. (**B**) A pie chart displaying the number and proportion of CCGs (*p* < 0.05, based on the Spearman correlation). (**C**) PCA of changes in CCGs after different refrigeration durations. The abscissa represents the first principal component PC1, and the ordinate represents the second principal component PC2. (**D**) KEGG enrichment analysis, where the x-axis represents the proportion of CCGs in each pathway among all CCGs, and the y-axis represents the name of the identified pathway. The size of the circles indicates the number of CCGs in each metabolic pathway, and their color indicates the *p* value from the concentration analysis.

The changes in CMCase activity were analyzed via a Spearman correlation analysis with transcriptomic data, and a total of 148 genes correlated with carboxymethyl cellulase were identified (*p* ≤ 0.05). The expression levels of these genes on days 0, 20, 40, 60, 80, and 100 are shown as heatmaps in Figure 3A. Among the 514 carboxymethyl cellulase-correlated genes (CCGs), 63.04% of the metabolites were positively correlated with the change in CMCase activity, and 36.96% showed the opposite correlation (Figure 3B). PCA of the observed gene expression levels revealed that the samples collected on days 40 and 60 were relatively similar (R^2^ = 0.982), and the six samples could be clearly distinguished (Figure 3C). Among the 514 carboxymethyl cellulase-correlated genes (CCGs), 63.04% of the metabolites were positively correlated (*p* < 0.05) with the change in CMCase activity, and 36.96% showed the opposite correlation (*p* < 0.05) (Figure 3C). The top 25 significantly enriched metabolic pathways are shown in Figure 3D; these pathways included sulfur metabolism, folate biosynthesis, and starch and sucrose metabolism. 3.4 CCMs and CCGs are enriched in metabolic pathways.

**Figure 4:**
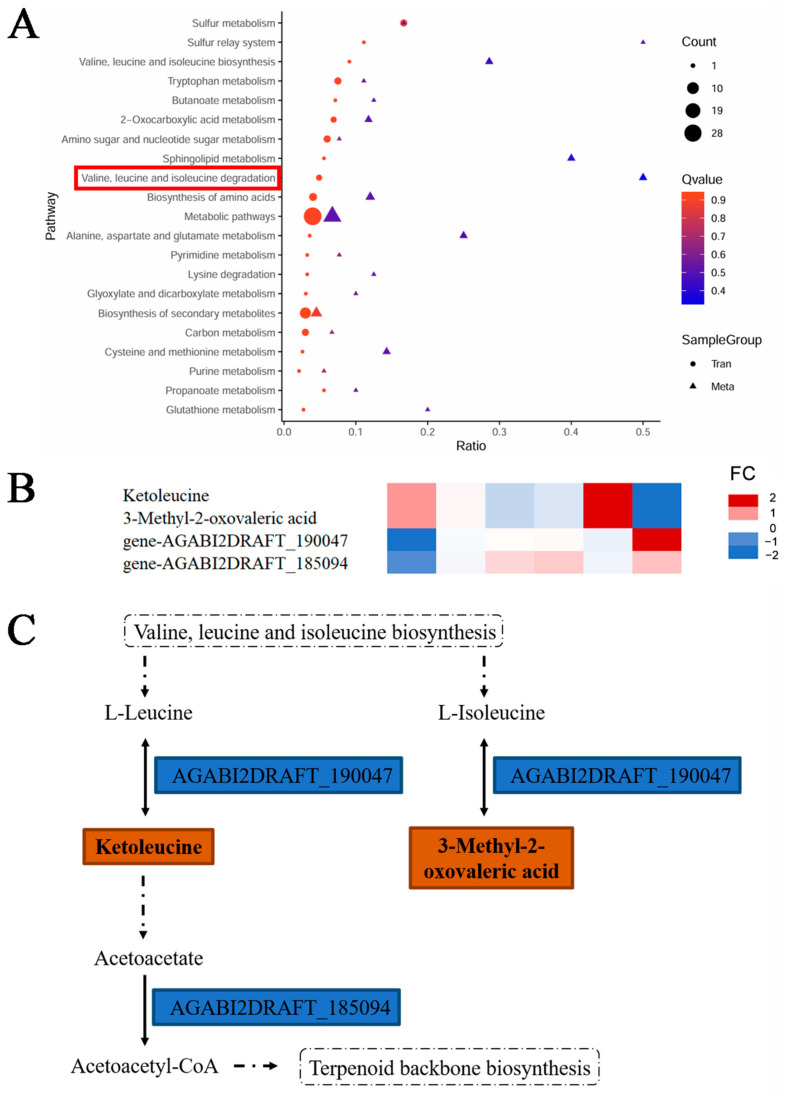

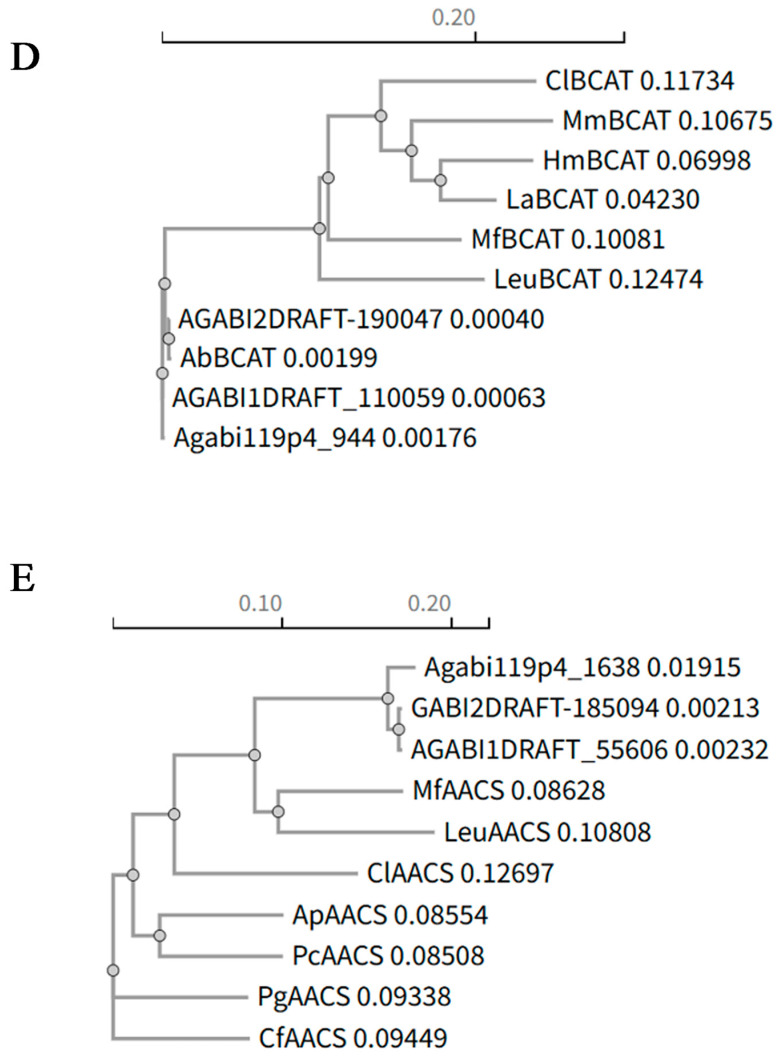
Combined transcriptomic and metabolomic analysis. (**A**) Joint KEGG analysis of CCMs and CCGs, where the x-axis represents the proportion of CCMs/CCGs in each pathway out of all CCMs/CCGs, and the y-axis represents the name of the identified pathway. The size of the circles indicates the number of CCMs/CCGs in each metabolic pathway, and their color indicates the *p* value from the concentration assay. The valine degradation pathway in the red box showed the strongest enrichment (*p* = 3.2 × 10^−5^), with consistent trends in both metabolites and genes. (**B**) Changes in the contents of CCMs and CCGs associated with valine, leucine, and isoleucine degradation. The FC represents the fold change. (**C**) Valine, leucine, and isoleucine degradation. The boxes represent DAMs and DEGs. (**D**) The phylogenetic analysis of *A. bisporus* AGABI2DRAFT-190047. (**E**) The phylogenetic analysis of *A. bisporus* AGABI2DRAFT-185094.

On the basis of the results for the CCMs and CCGs, a joint analysis of the metabolome and transcriptome was conducted to elucidate their relationships and regulatory mechanisms. The coenriched KEGG pathways from the combined analysis of DAMs and DEGs are shown in Figure 4A. The results revealed that metabolic pathways such as “metabolic pathways”, “biosynthesis of secondary metabolites”, and the valine degradation pathway showed the strongest enrichment (*p* = 3.2 × 10^−5^), with consistent trends in both metabolites and genes. Among these pathways, “valine, leucine, and isoleucine degradation” was the most prominent pathway in the combined analysis of CCMs and CCGs, involving 2 CCMs (ketoleucine and 3-methyl-2-oxovaleric acid) and 2 CCGs (AGABI2DRAFT-190047 and AGABI2DRAFT-185094).Comparative analysis of CCMs and CCGs revealed divergent expression patterns: two CCMs presented a gradual decrease in abundance, whereas two CCGs presented progressively increasing expression levels (Figure 4B). Figure 4C illustrates the functional roles of these two CCMs and two CCGs in the valine, leucine, and isoleucine degradation pathways. The constructed phylogenetic trees are shown in Figure 4D,E. The AGABI2DRAFT_190047 was identified as a homolog of BCAT and thus named AbbBCAT, while the gene AGABI2DRAFT_185094 was a homolog of AACS and designated AbbAACS. Specifically, the AbbBCAT regulates ketoleucine production through the valine, leucine, and isoleucine degradation pathway, and the AbbAACS mediates the conversion of the ketoleucine downstream product acetoacetate into acetoacetyl-CoA.

## 4. Discussion

The aging of food is always accompanied by changes in metabolites. Metabolic fluxes changed in *A. bisporus* cultivated spawn after refrigeration for different durations. The metabolites all changed significantly with increasing refrigeration time (Figure 1B and Figure 2A). These findings indicate that the direction of metabolic flux in cultivated *A. bisporus* spawn differed among different refrigeration durations. The mechanism of variation in the *A. bisporus* storage process has been widely confirmed in previous studies. The interactions between gene expression and metabolites are mutually reinforcing. Metabolites, in turn, are affected by genetically encoded proteins.

The activities of carboxymethyl cellulase, amylase, and acid protease decreased first but then increased, reaching the lowest level at approximately 40–60 days of storage and then increasing after 60–100 days (Figure 1A). It is possible that low-temperature refrigeration slows the metabolic rate of mycelia and decreases the activity of extracellular enzymes. However, at low temperatures, the mycelia still maintained a low rate of metabolism, and after storage at low temperatures for 40–60 days, the intracellular nutrients of the mycelia were slowly depleted, extracellular nutrients were needed, and the secretion of extracellular enzymes and enzyme activity increased [6,17]. When the laccase was stored at 8 °C for 0–100 days, the laccase enzyme activity first increased, peaking at approximately 40 days, and then gradually decreased from days 40 to 100 (Figure 1A). The medium (wheat kernels) of the mycelia may be interstitial, and the mycelia may not have entered the wheat grains. The main role of fungal laccases is to degrade the lignin components of the culture medium. The main components of wheat skin are cellulose and lignin encapsulated in cellulose. Although the overall metabolic rate is low, nutrients still need to be absorbed from the external environment. After lignin is degraded, it begins to gradually degrade the cellulose in the outer skin of the wheat grain, and as the mycelia slowly invade the inner layer of the wheat grain, the starch of the wheat endosperm can be used [6,18]. Malondialdehyde (MDA) is a product of cell membrane lipid peroxidation, reflecting the degree of oxidative damage to cellular structures. Higher MDA accumulation indicates more severe structural damage to cells caused by oxidation. During storage, the MDA content gradually increased over time. Compared with those stored at 4 °C and 1 °C, the mushrooms stored at 8 °C presented significantly higher MDA levels, with rates of increase being 6.28 times and 2.63 times faster, respectively. In contrast, the MDA levels in the mushrooms stored at 4 °C fluctuated only slightly throughout the storage period [19]. Thus, the cultivated spawn of *Agaricus bisporus* were stored at 8 °C in this study.

The composition of free amino acids in food contributes to characterizing its freshness and quality during storage. Different preservation methods for edible mushrooms (freezing, canning, and salting) lead to a reduction in the content of free amino acids. However, among the preservation methods for edible mushroom species, freezing has the least impact on the decrease in amino acids [20]. Furthermore, in studies on the effectiveness of maintaining the integrity and appearance of edible mushroom fruiting bodies, valine, leucine, and isoleucine have been identified as potential indicators for measuring the loss of brightness in edible mushrooms [21,22,23]. Research has shown that valine, leucine, and isoleucine alter the external color and morphology of edible mushroom cultivation spawn by influencing melanin formation in melanoma cells [22]. These reactions are closely related to quality damage and browning in agricultural products [24]. Therefore, we propose that the degradation of valine, leucine, and isoleucine is one of the main factors contributing to the aging of cultivated edible mushroom spawn during the refrigeration of cultivated *A. bisporus* spawn [25]. In this study, we hypothesize that enzymes and metabolites involved in the degradation of valine, leucine, and isoleucine may also affect the mycelial growth of cultivated *A. bisporus* spawn during refrigeration.

For example, the AbbBCAT governs ketoleucine production through the valine, leucine, and isoleucine degradation pathway. The downstream metabolite of ketoleucine, acetoacetate, serves as a crucial bioactive component in food microorganisms, influencing microbial activity and functioning as a potential nutritional metabolite that plays a vital role in the cold storage preservation of *Agaricus bisporus*. Moreover, AbbAACS modulates terpenoid backbone biosynthesis by regulating acetoacetyl-CoA production. Fungal terpenoids possess antioxidant properties that are particularly beneficial for low-temperature preservation. These findings may indicate that during the storage of cultivated *A. bisporus* spawn, the presence of ketoleucine and acetoacetate may serve as important biomarkers for evaluating refrigeration viability [26,27].

This study lays the groundwork for further investigations into the molecular mechanisms of cold adaptation in *A. bisporus* cultivation spawn. Future research should employ genetic approaches to validate key regulatory genes and expand multiomics analyses across temperature gradients. The identified metabolic signatures present opportunities for developing practical biomarkers to optimize industrial storage protocols. By combining multiomics approaches with enzymatic activity profiling, the work establishes a comprehensive framework linking molecular changes to physiological responses. These findings advance fundamental understanding of fungal cold adaptation while providing practical insights for optimizing industrial storage protocols, representing an important bridge between basic research and agricultural applications.

## 5. Conclusions

*A. bisporus* is the most popular mushroom in the world and has the most promising market potential. This study explored the physiological and biochemical changes that occur during refrigeration and provided new insights for the study of *A. bisporus* cultivation spawn storage methods. The results of this study revealed that four extracellular enzymes, namely, carboxymethyl cellulase, amylase, acidic protease, and laccase, indirectly promote activity by influencing the utilization of the culture medium for spawn mycelium growth. The regulation of transcription factors and metabolites associated with valine, leucine, and isoleucine degradation affected the quality of cultivated *A. bisporus* spawn during storage. The findings of this study suggest that storage beyond 60 days at 8 °C leads to significant metabolic changes that may impact spawn viability, for industrial applications, spawn storage should not exceed 60 days at 8 °C to avoid viability loss. While this study focused on molecular-level changes under refrigeration duration; future work should incorporate sensory evaluation to correlate metabolic changes with product quality, for example, validation of ketoleucine as a biomarker and sensory evaluations are needed. And interactions with humidity, temperature, and ventilation condition gradients to optimize storage protocols should be explored further.

## Data Availability

The original contributions presented in this study are included in the article. Further inquiries can be directed to the corresponding author.
